# Supplementary material for: A benzodiazepine activator locks Kv7.1 channels open by electro-mechanical uncoupling
Source: Commun Biol. 2022 Apr 1;5:301. doi: 10.1038/s42003-022-03229-8 (PMC8976019; doi:10.1038/s42003-022-03229-8)
Supplement: Supplementary file 2 — Supplementary information [file 42003_2022_3229_MOESM2_ESM.pdf]

Supplementary Information

A Benzodiazepine Activator Locks $K_V7.1$ Channels Open by Electro-Mechanical Uncoupling

Running title: RL3 Binding and Activation

Julian A. Schreiber[1,2,*], Melina Möller[1,*], Mark Zaydman[3], Lu Zhao[3], Zachary Beller[3], Sebastian Becker[1], Nadine Ritter[1,4], Panpan Hou[3], Jingyi Shi[3], Jon Silva[3], Eva Wrobel[1], Nathalie Strutz-Seebohm[1], Niels Decher[5], Nicole Schmitt[6], Sven G. Meuth[7], Martina Düfer[2,4], Bernhard Wünsch[2,4], Jianmin Cui[3] and Guiscard Seebohm[1,4,π]

[1]*Institute for Genetics of Heart Diseases (IfGH), Department of Cardiovascular Medicine, University Hospital Münster, D-48149 Münster, Germany.*

[2]*Institute of Pharmaceutical and Medicinal Chemistry, University of Münster, Corrensstr. 48, Münster, D-48149, Germany.*

[3]*Department of Biomedical Engineering, Center for the Investigation of Membrane Excitability Disorders, Cardiac Bioelectricity and Arrhythmia Center, Washington University, St. Louis, MO 63130, United States of America.*

[4]*Chembion, University of Münster, D-48149 Münster, Germany.*

[5]*Institute of Physiology and Pathophysiology, Vegetative Physiology, Philipps-University of Marburg, Deutschhausstr. 1-2, 35037 Marburg, Germany.*

[6]*Department of Biomedical Sciences, University of Copenhagen, Copenhagen, Denmark.*

[7]*Department of Neurology, University Hospital Düsseldorf, Düsseldorf, Germany.*

* These authors contributed equally.

[π] Corresponding author:
Prof. Dr. Guiscard Seebohm
email: guiscard.seebohm@ukmuenster.de

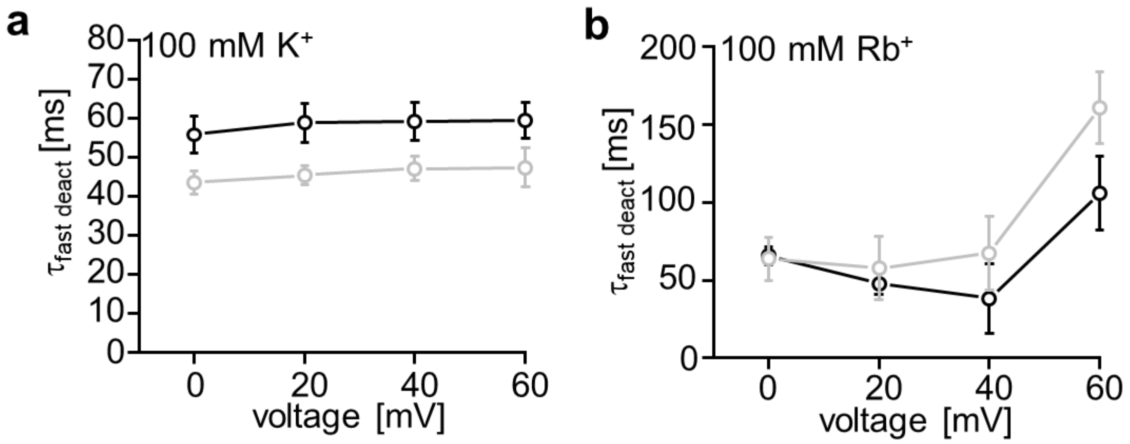

SI Figure 1 Fast deactivation component of Kv7.1 in high K^+. (**a**) and high Rb^+ (**b**) in absence (black) and presence (gray) of (*R*)-L3. Time constants $\tau_{fast\ deact}$ were determined by two-component exponential fit for each oocyte and voltage step and are given as mean ± SEM from n=15-18 oocytes. Significance of mean differences were evaluated by one-way ANOVA and posthoc mean comparison Tukey test. However, no significant differences were observed.

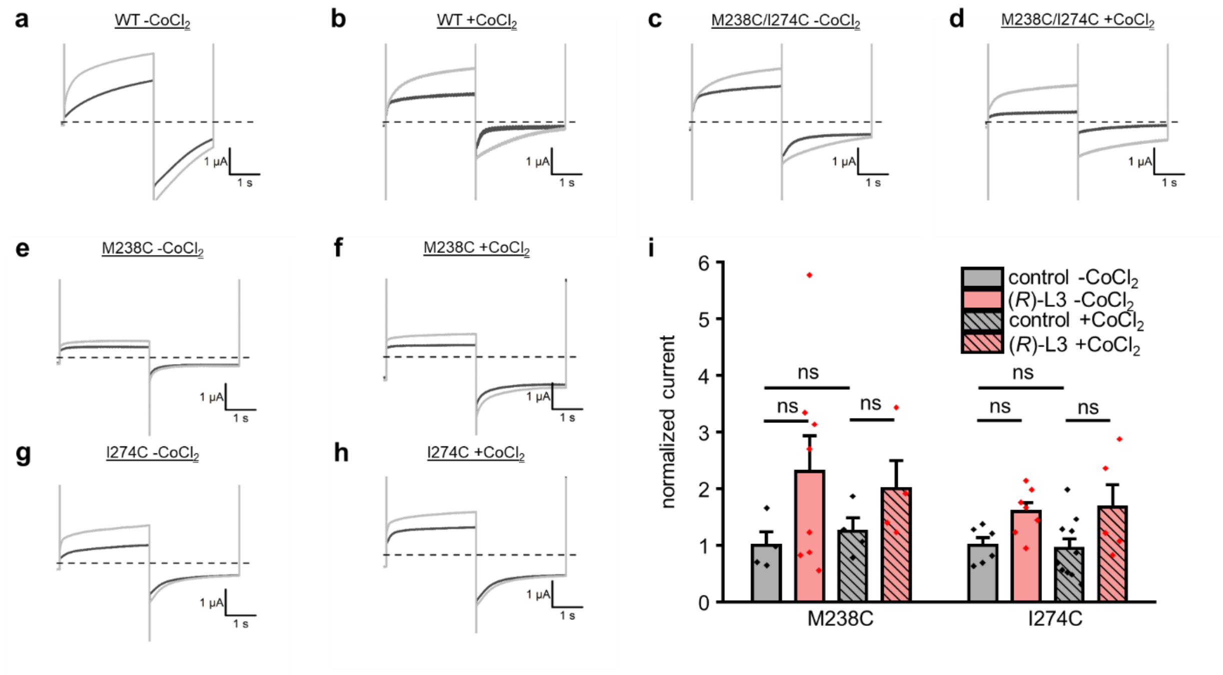

SI Figure 2 Sample traces and normalized currents for K_v7.1 WT, Kv7.1 M238C, K_v7.1 I274C and K_v7.1 M238C/I274C in absence/presence of (*R*)-L3 and $CoCl_2$. a-h sample traces for wildtype, single and double mutants in presence (gray) and absence (black) of (*R*)-L3 as well as presence/absence of $CoCl_2$. **i** Normalized currents of single mutants Kv7.1 M238C and K_v7.1 I274C. currents were normalized to the current of the same mutant in absence of (*R*)-L3 and $CoCl_2$. Significance of mean differences were evaluated by one-way ANOVA and posthoc mean comparison Tukey test (ns for $p > 0.05$).

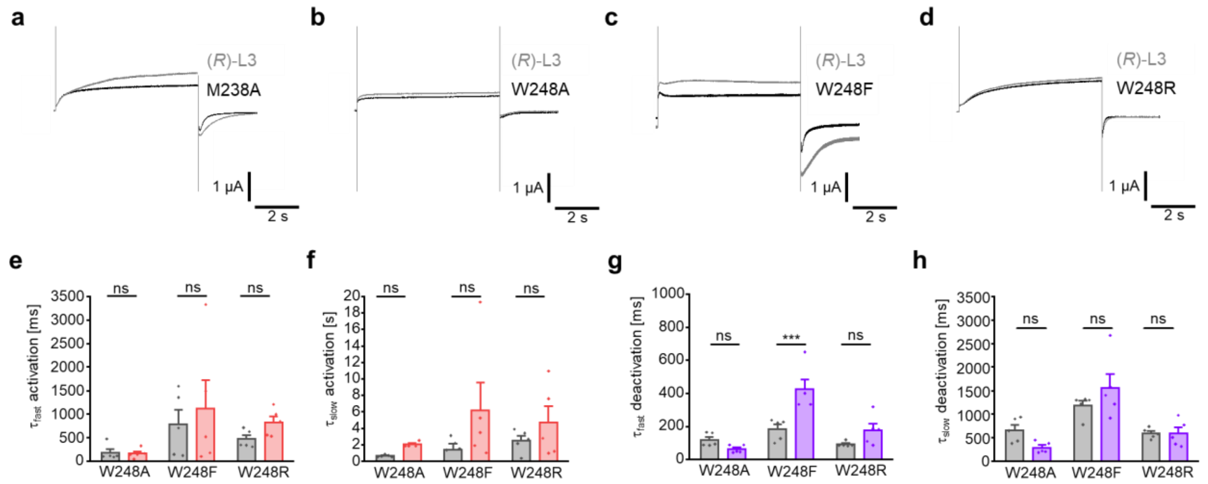

SI Figure 3 Sample traces and kinetic parameters of W248 mutants. a-d. Sample traces for $K_V7.1$ M238A, W248A, W248F and W248R and kinetic parameters of W248 mutants. **e-h**. Time constants for fast and slow activation and deactivation. Time constants were determined by two-component exponential fit. Significance of mean differences were evaluated by one-way ANOVA and posthoc mean comparison Tukey test (ns for $p > 0.05$; *** for $p < 0.001$).

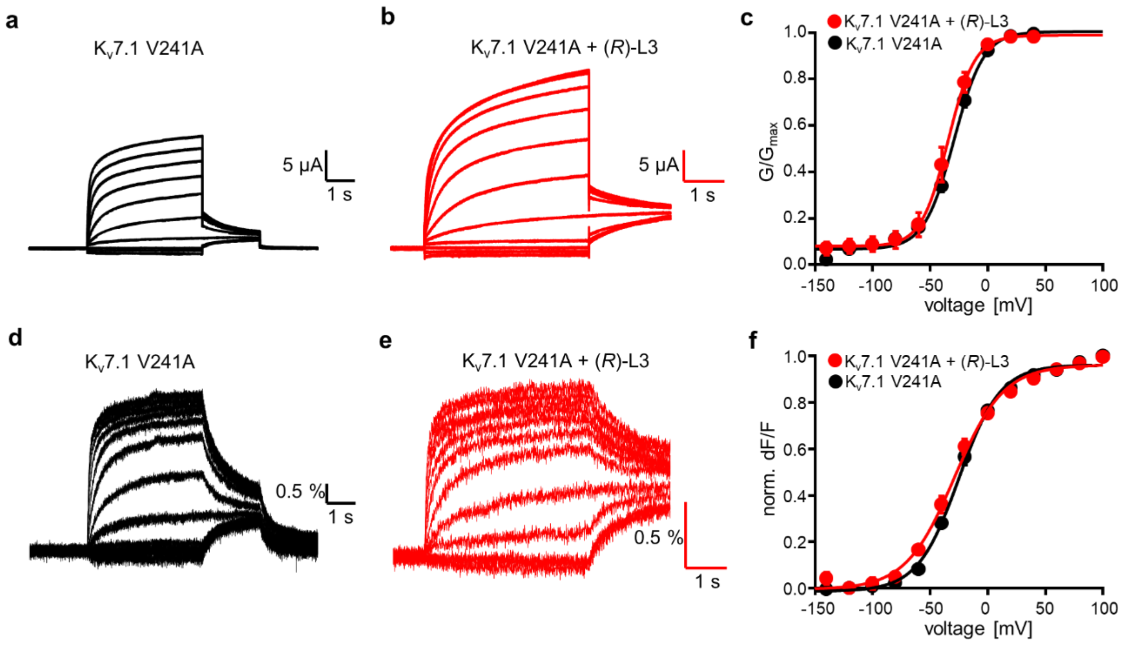

SI Figure 4 VCF results for $K_v7.1_{VCF}$ V241A. a-b Current sample traces for $K_v7.1_{VCF}$ V241A expressing oocytes in absence (**a**) and presence (**b**) of (*R*)-L3. **c** G/G_{max} relationship for $K_v7.1$ V241A expressing oocytes in absence (black) and presence (red) of (*R*)-L3. Normalized currents were fitted by Boltzmann equation. **d-e** Fluorescence sample traces for $K_v7.1_{VCF}$ V241A expressing oocytes in absence (**d**) and presence (**e**) of (*R*)-L3. **f** dF/F relationship for $K_v7.1_{VCF}$ V241A expressing oocytes in absence (black) and presence (red) of (*R*)-L3. Normalized fluorescence data were fitted by Boltzmann equation.

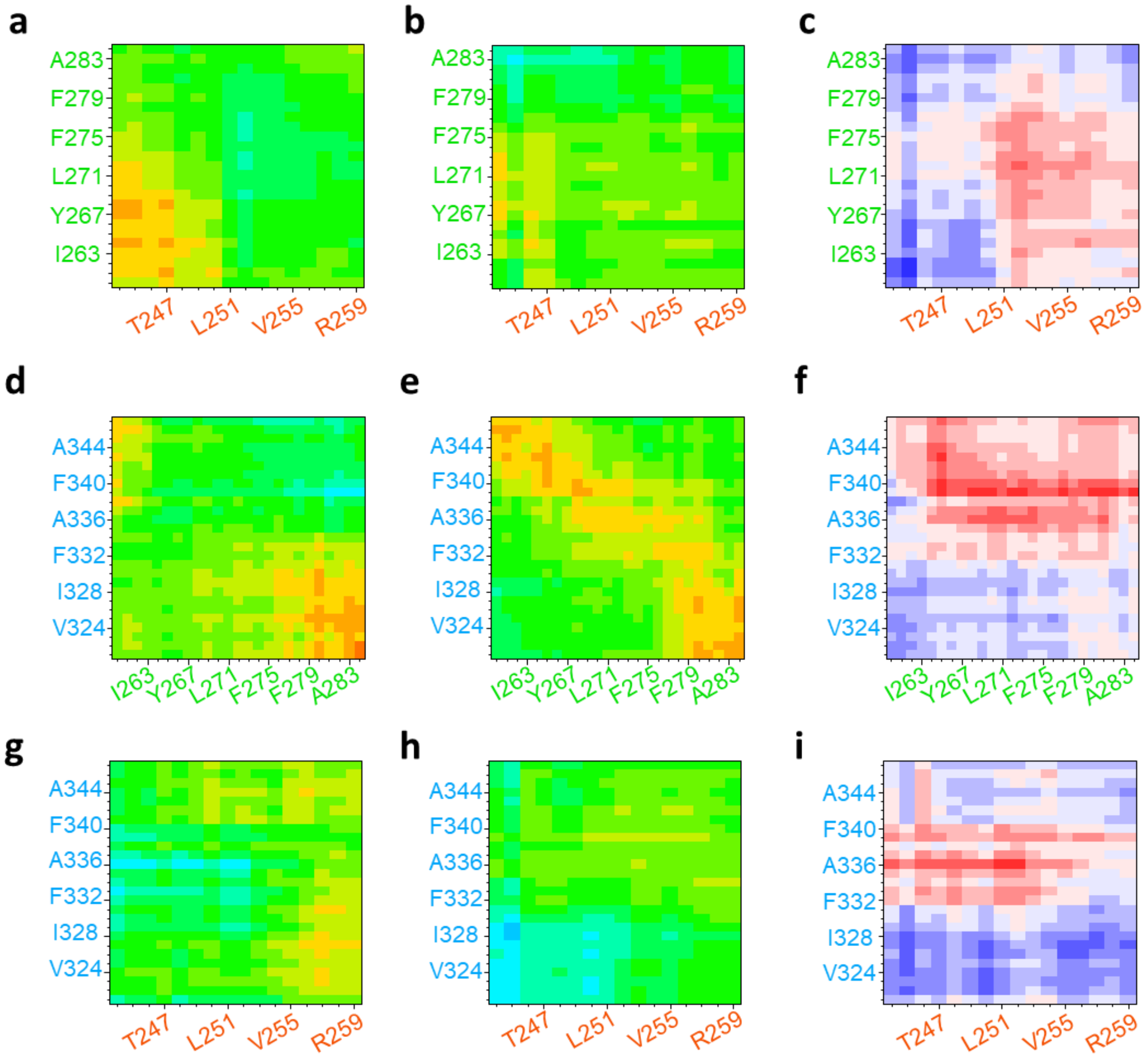

SI Figure 5 DCCM matrices. **a-b** Dynamic cross correlation matrix (DCCM) for S5 (Mol A, green) and S4S5 linker (Mol B, orange) in absence (**a**) and presence (**b**) of (*R*)-L3 from -1 (fully anticorrelated) over 0 (not correlated) to 1 (fully correlated). **c** Increase (positive values, red) and decrease (negative values, blue) of correlation between S5 and S4S5 linker residues depending on presence of (*R*)-L3. **d-e** DCCM for S6 (Mol A, blue) and S5 (Mol A, green) in absence (**d**) / presence (**e**) of (*R*)-L3. **f** Increase (positive values, red) and decrease (negative values, blue) of correlation between S6 and S5 residues depending on presence of (*R*)-L3. **g-h** DCCM for S6 (Mol A, blue) and S4S5 linker (Mol B, orange) in absence (**g**) / presence (**h**) of (*R*)-L3. **i** Increase (positive values, red) and decrease (negative values, blue) of correlation between S6 and S4S5 linker residues depending on presence of (*R*)-L3.

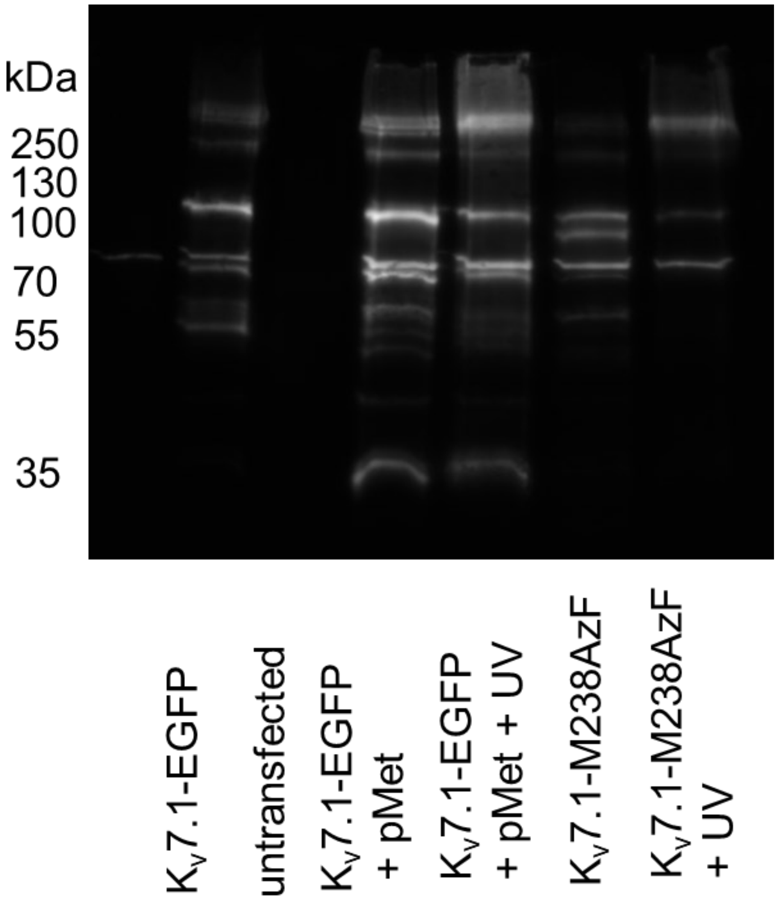

SI Figure 6 Uncropped and unedited blot image for main Figure 5e.

SI Table 1 number of independent oocytes (n) expressing $hK_v7.1$ variants for kinetic measurements in Figure 4 in absence/presence of (*R*)-L3.

Figure	(*R*)-L3	WT	I235A	L236A	R237A	M238A	L239A	H240A	V241A
4f	without	11	18	13	-	14	11	14	13
	with	11	17	16	-	14	11	13	14
4g	without	10	14	13	15	14	12	11	19
	with	10	16	10	13	14	12	12	15
4h	without	8	17	13	-	12	9	14	18
	with	8	17	13	-	12	9	12	18
4i	without	8	18	15	11	14	11	14	17
	with	8	15	11	12	14	11	11	17

SI Table 2 number of independent oocytes (n) expressing $hK_v7.1$ variants for Co^{2+} crosslinking measurements in Figure 5 and SI Figure 2 in absence/presence of (*R*)-L3 and Co^{2+}.

(*R*)-L3	Co^{2+}	WT	M238C	I274C	M238C / I274C
without (ctrl)	without	14	4	6	19
with	without	17	8	7	19
without (ctrl)	with	14	4	10	24
with	with	14	4	5	23

SI Table 3 Parameters of MD Simulations.

**Used structures**	Homology models published by Kuenze et al.[1]
**Box size**	AO 125.87 x 88.84 x 125.87 Å<br>AC 131.26 x 96.67 x 131.26 Å<br>RC 118.27 x 91.30 x 118.27 Å
**Total number of atoms**	AO 138,130 atoms + 51 for (*R*)-L3<br>AC 161,741 atoms + 51 for (*R*)-L3<br>RC 125,166 atoms + 51 for (*R*)-L3
**Number of water molecules**	AO 27,073 molecules<br>AC 33,426 molecules<br>RC 25,232 molecules
**Water model**	AO TIP3P<br>AC TIP3P<br>RC TIP3P
**Salt concentration**	AO 0.9 % NaCl<br>AC 0.9 % NaCl<br>RC 0.9 % NaCl
**Number of ions**	AO 182 (Na^+, Cl^-)<br>AC 233 (Na^+, Cl^-)<br>RC 166 (Na^+, Cl^-)

Supplementary References

[1] G. Kuenze, A. M. Duran, H. Woods, K. R. Brewer, E. F. McDonald, C. G. Vanoye, A. L. George, C. R. Sanders, J. Meiler, *PLoS One* **2019**, *14*, e0220415.
