# Supplementary material for: A benzodiazepine activator locks Kv7.1 channels open by electro-mechanical uncoupling
Source: Commun Biol. 2022 Apr 1;5:301. doi: 10.1038/s42003-022-03229-8 (PMC8976019; doi:10.1038/s42003-022-03229-8)
Supplement: Supplementary file 3 — Description of Additional Supplementary Files [file 42003_2022_3229_MOESM3_ESM.pdf]

Description of Additional Supplementary Files

File name: Supplementary Data
Description: Source data for graphs and charts.
