# Supplementary material for: A benzodiazepine activator locks Kv7.1 channels open by electro-mechanical uncoupling
Source: Commun Biol. 2022 Apr 1;5:301. doi: 10.1038/s42003-022-03229-8 (PMC8976019; doi:10.1038/s42003-022-03229-8)
Supplement: Supplementary file 5 — Reporting Summary [file 42003_2022_3229_MOESM5_ESM.pdf]

nature portfolio

Corresponding author(s): Prof. Dr. Guiscard Seebohm

Last updated by author(s): Jan 21, 2022

Reporting Summary

Nature Portfolio wishes to improve the reproducibility of the work that we publish. This form provides structure for consistency and transparency in reporting. For further information on Nature Portfolio policies, see our Editorial Policies and the Editorial Policy Checklist.

Statistics

For all statistical analyses, confirm that the following items are present in the figure legend, table legend, main text, or Methods section.

n/a	Confirmed	
☐	☒	The exact sample size (*n*) for each experimental group/condition, given as a discrete number and unit of measurement
☐	☒	A statement on whether measurements were taken from distinct samples or whether the same sample was measured repeatedly
☐	☒	The statistical test(s) used AND whether they are one- or two-sided<br>*Only common tests should be described solely by name; describe more complex techniques in the Methods section.*
☒	☐	A description of all covariates tested
☒	☐	A description of any assumptions or corrections, such as tests of normality and adjustment for multiple comparisons
☐	☒	A full description of the statistical parameters including central tendency (e.g. means) or other basic estimates (e.g. regression coefficient) AND variation (e.g. standard deviation) or associated estimates of uncertainty (e.g. confidence intervals)
☒	☐	For null hypothesis testing, the test statistic (e.g. F, t, r) with confidence intervals, effect sizes, degrees of freedom and P value noted<br>*Give P values as exact values whenever suitable.*
☒	☐	For Bayesian analysis, information on the choice of priors and Markov chain Monte Carlo settings
☒	☐	For hierarchical and complex designs, identification of the appropriate level for tests and full reporting of outcomes
☒	☐	Estimates of effect sizes (e.g. Cohen's d, Pearson's r), indicating how they were calculated

Our web collection on statistics for biologists contains articles on many of the points above.

Software and code

Policy information about availability of computer code

Data collection	For data collection GePulse software (Michael Pusch, Genova, Italy) and YASARA 19 (YASARA Biosciences GmbH, Vienna, Austria) were used.
Data analysis	Data analysis was performed using Ana (Michael Pusch, Genova, Italy), GraphPad Prism 5.01 (GraphPad Software, San Diego, California) and OriginPro2020.

For manuscripts utilizing custom algorithms or software that are central to the research but not yet described in published literature, software must be made available to editors and reviewers. We strongly encourage code deposition in a community repository (e.g. GitHub). See the Nature Portfolio guidelines for submitting code & software for further information.

Data

Policy information about availability of data

All manuscripts must include a data availability statement. This statement should provide the following information, where applicable:

- Accession codes, unique identifiers, or web links for publicly available datasets
- A description of any restrictions on data availability
- For clinical datasets or third party data, please ensure that the statement adheres to our policy

All data are available on reasonable request.

Field-specific reporting

Please select the one below that is the best fit for your research. If you are not sure, read the appropriate sections before making your selection.

☒ Life sciences ☐ Behavioural & social sciences ☐ Ecological, evolutionary & environmental sciences

For a reference copy of the document with all sections, see nature.com/documents/nr-reporting-summary-flat.pdf

Life sciences study design

All studies must disclose on these points even when the disclosure is negative.

Sample size	Each experiment was perfomed with a minimum sample size of 3 independent measurements or 3 independend simulations.
Data exclusions	Wild type and mutant channels expressed in Xenopus laevis were only used if the current at 40 mV was ≥0.5 µA, which is robust enough for analyses.
Replication	Each experiment was perfomed with a minimum sample size of 3 independent measurements or 3 independend simulations.
Randomization	Samples were grouped by parameters like expressed Kv7.1 variant, absence/presence of (R)-L3, etc. Grouping parameters are indicated in the manuscript for each experiment.
Blinding	Blinding was not performed, since no data were collected from animal or human participants.

Reporting for specific materials, systems and methods

We require information from authors about some types of materials, experimental systems and methods used in many studies. Here, indicate whether each material, system or method listed is relevant to your study. If you are not sure if a list item applies to your research, read the appropriate section before selecting a response.

Materials & experimental systems

n/a	Involved in the study	
☐	☒	Antibodies
☐	☒	Eukaryotic cell lines
☒	☐	Palaeontology and archaeology
☒	☐	Animals and other organisms
☒	☐	Human research participants
☒	☐	Clinical data
☒	☐	Dual use research of concern

Methods

n/a	Involved in the study	
☒	☐	ChIP-seq
☒	☐	Flow cytometry
☒	☐	MRI-based neuroimaging

Antibodies

Antibodies used	For detection of Kv7.1 monoclonal Kv7.1 antibody (sc-365186, Santa Cruz Biotechnology) was used.
Validation	See monoclonal Kv7.1 antibody (sc-365186) from Santa Cruz Biotechnology.

Eukaryotic cell lines

Policy information about cell lines

Cell line source(s)	HEK293T were purchased from Sigma-Aldrich.
Authentication	HEK293T were purchased from Sigma-Aldrich.
Mycoplasma contamination	Cells had no mycoplasma contamination.
Commonly misidentified lines (See ICLAC register)	not applicable.
